# Essential Oil Analysis and Antimicrobial Evaluation of Three Aromatic Plant Species Growing in Saudi Arabia

**DOI:** 10.3390/molecules26040959

**Published:** 2021-02-11

**Authors:** Hamdi El-Said, Sami S. Ashgar, Ammar Bader, Aljawharah AlQathama, Majed Halwani, Roberta Ascrizzi, Guido Flamini

**Affiliations:** 1 Department of Medical Microbiology, Faculty of Medicine, Umm Al-Qura University, Makkah 21955, Saudi Arabia; hmibrahim@uqu.edu.sa (H.E.-S.); ssashgar70@hotmail.com (S.S.A.); 2 Department of Pharmacognosy, Faculty of Pharmacy, Umm Al-Qura University, Makkah 21955, Saudi Arabia; aaqathama@uqu.edu.sa; 3 King Abdullah International Medical Research Center, King Saud Bin Abdulaziz University for Health Sciences, Riyadh 11481, Saudi Arabia; halawanima@ngha.med.sa; 4 Department of Pharmacy, University of Pisa, Via Bonanno 6, 56126 Pisa, Italy; roberta.ascrizzi@gmail.com

**Keywords:** carvacrol, *Lavandula pubescens*, *Pulicaria incisa*, *Juniperus procera*, holy sites health

## Abstract

Arabian flora is a rich source of bioactive compounds. In this study, we investigated three aromatic plant species with the aim of finding valuable sources of antimicrobial agents against common pathogenic microorganisms. We focused especially on microorganisms, which cause outbreaks of infectious disease during mass gatherings and pilgrimages season in Saudi Arabia. The essential oils of three aromatic plant species were hydrodistilled from flowering aerial parts of *Lavandula pubescens* Decne. and *Pulicaria incisa* subsp. *candolleana* E.Gamal-Eldin, and from leaves, stems, ripe and unripe fruits of *Juniperus procera* Hochst. Ex Endl. They were subsequently analyzed by gas chromatography-mass spectrometry (GC-MS). The main constituents of *L. pubescens* were found to be carvacrol (55.7%), methyl carvacrol (13.4%), and β-bisabolene (9.1%). *P. incisa* subsp. *Candolleana* essential oil was rich in linalool (33.0%), chrysanthenone (10.3%), eugenol (8.9%), and *cis*-chrysanthenol (8.0%); the major components of *J. procera* essential oil were α-pinene (31.3–62.5%) and δ-3-carene (7.3–30.3%). These essential oils were tested against thirteen American Type Culture Collection (ATCC) strains of Gram-positive and Gram-negative bacteria using the agar diffusion assay. The only effective essential oil was that of *L. pubescens* and the most sensitive strains were *Acinetobacter baumannii*, *Salmonella typhimurium, Shigella sonnei*, *Enterococcus faecalis* and *Staphylococcus epidermidis*. Carvacrol, the major constituent of *L. pubescens*, was tested on these strains and was compared with vancomycin, amikacin, and ciprofloxacin. The Minimum Inhibitory Concentration (MIC) and Minimum Bactericidal Concentration (MBC) assays of *L. pubescens* essential oil and carvacrol revealed that Gram-negative strains were more susceptible than the Gram-positive ones.

## 1. Introduction

Plants represent valuable sources of bioactive molecules belonging to various classes of secondary metabolites. Among the physiological roles of secondary metabolites in plants, is the resistance to phytopathogens, including bacteria, fungi and viruses [1,2,3]. The majority of these metabolites have the ability to interact with cellular enzymes or cell structure, causing irreversible damage to the invasive microorganisms [4,5,6]. For this reason, plant secondary metabolites have become an interesting target for the discovery of new bioactive molecules with antimicrobial effects and variable modes of action, especially after the recent emergence and growth of antibiotic resistance. According to the World Health Organization (WHO), the current rise in antibiotic resistance is due to the misuse of pharmaceutical antibiotics and is a major cause of the prolongation of illness with higher risk of death [7].

Essential oils (EOs) have numerous commercial applications due to their diverse biological properties and appealing fragrances. The global market thus supports a wide variety of pharmaceutical products such as gels, creams, ointments, nano-emulsions, and patches [8,9,10,11]. Besides their exceptional antimicrobial effects, they also possess a wide range of pharmacological properties, including, for example, analgesic, anti-inflammatory, antidiabetic, anti-parasitic, anticancer and antioxidant activity [12,13,14,15,16]. The use of EOs in therapy has increased in recent times due to the rise in multidrug-resistant bacteria and the high costs of new generation antibiotic drugs [17]. The chemical nature of essential oils hinders the process of microbial resistance, since they are very complex mixtures of constituents with different structures, including monoterpenes [18], sesquiterpenes [19], diterpenes [20], sulfur-containing compounds [21,22], phenylpropanoids [20], alkaloids [23], and phenols [24]. This large variety of chemical structures means that no single enzyme is able to deactivate all of these compounds. Thus, EOs may represent an open frontier for advances in medicine and pharmaceutical sciences.

The flora of Saudi Arabia is very rich and variable due to environmental diversity, ranging from extreme arid desert to high mountains with high rainfall rates. These variations affect plant metabolism in terms of secondary metabolite chemistry and biological activity. In fact, some Saudi plants have been found to exhibit substantial chemo-diversity from the same plants grown in other countries and climates [25,26,27]. The aim of this study was to investigate the chemical composition of hydrodistilled EOs from different organs of three aromatic plant species collected in Saudi Arabia and to find valuable sources of antimicrobial agents against common pathogenic microorganisms. These were the aerial parts of *Lavandula pubescens* Decne. and *Pulicaria incisa* subsp. *candolleana* E.Gamal-Eldin, as well as leaves, stems, ripe and unripe fruits of *Juniperus procera* Hochst. Ex Endl.

*L. pubescens* is reported in the literature as a species commonly found among the wild flora in Middle-Eastern Asia and Mediterranean Africa; it is, indeed, mainly reported as growing in Palestine [28], Yemen [29,30,31], Saudi Arabia [32,33,34]. For *P. incisa* subsp. *Candolleana*, to the best of our knowledge, only one study is reported in the literature, analyzing wild-growing specimens in Egypt [35]. However, *Pulicaria* spp. are reported as widely used in Northern African folk-medicine [36]. These species have been chosen for their good availability in the wild, which makes them an exploitable and easy to gather biomass in their native range.

Recently, in Saudi Arabia, new outbreaks of multidrug-resistant pathogenic microorganisms have been recorded in intensive care units, including Gram-positive and Gram-negative bacteria, out of which *Acinetobacter baumannii*, *Pseudomonas aeruginosa, Escherichia coli,* and *Klebsiella pnemoniae* can cause fatal respiratory tract infections and pneumonia, blood stream infections and urinary tract infections. Not only this, but also the transmission and spread of infectious diseases during mass public gatherings such as Hajj (an annual pilgrimage to the Holy Mecca, Saudi Arabia) poses an enormous challenge. The global spread of antibiotic-resistant bacteria by international travelers may occur during pilgrimages or when visitors return to their home countries [37]. The pathogenic microorganisms mentioned above have gradually become less susceptible to a broad spectrum of potent antibiotics such as imipenem, meropenem, ciprofloxacin, amikacin and cefuroxime [38]. Due to the increasing presence of multidrug-resistant pathogenic microorganisms, the present study also aims to test these three EOs against three Gram-positive and Gram-negative bacteria to assess their potential use as alternative antimicrobial agents.

## 2. Results and Discussion

### 2.1. Essential Oil Compositions

The six hydrodistilled EOs were analyzed by GC-MS; their complete compositions and hydrodistillation yields are reported in Table 1.

**Figure 1:**
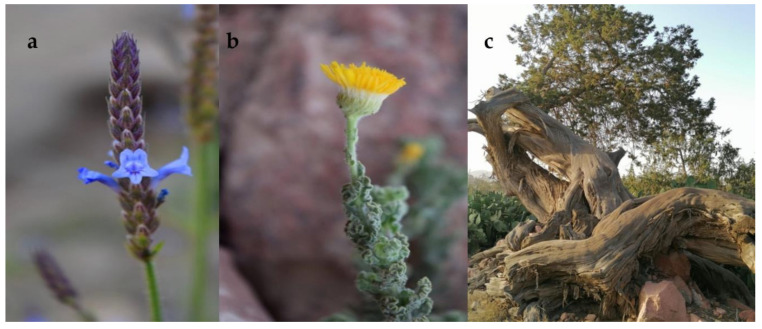
(**a**) *Lavandula pubescens* flowering aerial part; (**b**) *Pulicaria incisa* ssp. *candolleana* flowering aerial part; (**c**) *Juniperus procera*.

The EO of *L. pubescens* (Figure 1a) revealed a total of 19 different compounds. Oxygenated monoterpenes were the predominant class, accounting for 70.1% of the whole oil. Among them, carvacrol (55.7%) and methyl carvacrol (13.4%) were the main constituents. Monoterpene hydrocarbons were the second most abundant class, which included terpinolene (6.1%), *(Z)*-β-ocimene (4.1%) and myrcene (3.5%) among the most represented compounds. Sesquiterpene hydrocarbons accounted for 13.1%, with β-bisabolene as the most abundant (9.1%). The predominance of carvacrol and methyl carvacrol as the most abundant compounds in the *L. pubescens* EO of this study is in accordance with published compositions of the EOs hydrodistilled from several Yemeni specimens [29,30]. This species is, indeed, reported as having a phenolic-type EO profile among species belonging to this genus [39]. Several bioactivities of the EO are reported in the literature; for example, it is antioxidant and antimicrobial towards a wide variety of bacterial and fungal strains responsible for human and animal diseases, e.g., *Staphylococcus aureus*, *E. coli*, *Candida albicans*, and *Microsporum canis* [28,30].

In the EOs of *Pulicaria incisa* ssp. *candolleana* (Figure 1b)*,* 36 compounds were detected. Its EO was rich in oxygenated monoterpenes, accounting for 64.2% of the whole oil, with linalool (33.0%), chrysanthenone (10.3%), and *cis*-chrysanthenol (8.0%) as the main constituents. Phenylpropanoids represented the second most abundant class, with eugenol (8.9%) as the sole representative compound. The class of non-terpene derivatives accounted for 8.7%, including mainly (*Z*)-jasmone (4.7%) and isopentyl 2-methylbutanoate (2.6%). Only one previous study reported the composition of the EOs of *P. incisa* ssp. *candolleana* collected in Egypt, with carvotanacetone (66.01–50.87%) and chrysanthenone (13.26–24.3%) as the most representative components, of which the latter was also present in a significant percentage (10.3%) in our sample. EOs hydrodistilled from this Egyptian sample showed antimicrobial activity against Gram-positive and Gram-negative bacteria, as well as against some fungi, among which *Streptococcus pneumonia*, *E. coli* and *Syncephalastrum racemosum* were the most sensitive strains [35].

All EOs distilled from the different parts of *J. procera* (Figure 1c) were compared, revealing minor chemical differences. EO compositions showed some common compounds in all the investigated parts, such as α-pinene (31.3–62.5%), δ-3-carene (7.3–30.3%), α-humulene (1.5–6.9%), β-caryophyllene (1.6–6.4%), and β-pinene (3.3–4.6%). Ripe and unripe fruits showed the least differences, while the stems EO contained higher percentages of β-bisabolene (9.1%), which was completely absent in leaf and fruit EOs. A previous study on the fruit EO of *J. procera* reported the presence of eugenol as the main constituent (78.4%); although this phenylpropanoid was completely absent in our sample, these two compositions shared the presence of α-pinene and β-caryophyllene, even in different percentages [40]. The cited study, however, does not specify the ripeness stage of the hydrodistilled material. The EOs of the leaves reported in this study shared some constituents with Ethiopian samples, whose literature-reported EOs compositions showed α-pinene (28.1%), δ-3-carene (29.6%), β-pinene (4.35%), elemol (1.8%) and terpinolene (4.1%) [41,42]. A published study by Burits et al. (2001) on *J. Procera* leaf EO reported its antioxidant capacity [43].

### 2.2. Antimicrobial Activity of the Essential Oils

The EOs of *L. pubescens*, *P. incisa* ssp. *candolleana*, and *J. procera* leaves were tested against 13 different microbial strains, using diffusion assay on agar plates at the concentration of 20 µL per well (equivalent to 200 µg); vancomycin, amikacin (30 µg per disc), and ciprofloxacin were used as positive controls (Table 2). Only the EO of *L. pubescens* was significantly active, and, among all tested strains, only towards *Enterococcus faecalis* ATCC 51299 (inhibition zone 12 mm), *Staphylococcus epidermidis* ATCC 12228 (10 mm), *Salmonella typhimurium* ATCC 700720 (13 mm), *A. baumannii* (CRE) ATCC 1605 (15 mm), and *Shigella sonnei* ATCC 25931 (11 mm). *A. baumannii* has been reported by Haseeb et al. (2016) to be among the most common isolated Gram-negative pathogens, with high resistance rate to tobramycin, and *E. faecalis* and *S. epidermidis* are the most frequently reported Gram-negative pathogens during pilgrimage season [46]. In addition, antibiotic-resistant *S. sonnei* infections are commonly reported in mass gathering events [47]. Thus, finding antimicrobial activity in the EO of *L. pubescens* that works against these reported resistant pathogens will open the door to utilizing the essential oil of this plant as an alternative natural control against the spread of resistant pathogens during mass gatherings.

### 2.3. Antimicrobial Activity of L. Pubescens and Carvacrol

The EO of *L. pubescens* was tested at two concentrations (200 µg and 300 µg per well) to obtain better information about its efficacy in comparison with its most abundant constituent (carvacrol). The EO exerted better inhibitory results against *A. baumannii* (CRE) ATCC 1605, compared to those obtained with carvacrol. This increased efficacy could be a synergistic effect of other constituents of *L. pubescens* EO. When testing carvacrol, inhibition zones were more pronounced than those of the crude oil for *S. epidermidis* ATCC 12228 (15 mm) and *S. typhimurium* ATCC 700720 (20 mm), *A. baumannii* (CRE) ATCC 1605 (15 mm), and *S. sonnei* ATCC 25931 (15 mm). Gram-positive strains were more sensitive to the antibiotic vancomycin (30µg disc), showing more pronounced inhibition zones for *E. faecalis* (18.5 mm) and *S. epidermidis* (28 mm).

In the case of Gram-negative strains, the antibiotic amikacin (30µg disc) showed inhibition activity against *S. typhimurium* (20 mm) and *A. baumannii* (CRE) (15 mm), while *S. sonnei* was observed to be resistant. All strains were not affected by dimethyl sulfoxide (DMSO), which was used as a negative control (Table 3). The antimicrobial activity of carvacrol has been reported similarly by others against *E. faecalis* [48], *S. typhimurium* [49], and *A. baumannii* indicating its potential effects in infectious disease control; antibacterial and antibiofilm activity against *Salmonella enterica* serotype; antimicrobial activity of essential oils-derived volatile compounds against several nosocomial pathogens including representative multidrug-resistant *A. baumannii* clinical isolates [9].

As shown in Table 4, Gram-negative strains exhibited a higher susceptibility to both EO and carvacrol than the Gram-positive ones (Table 4).

As shown in Table 3 and Table 4, both carvacrol and *L. pubescens* EO could be promising candidates for the development of formulas to be used mainly for the treatment of intestinal diseases caused by *S. typhimurium* and *S. sonnei*. The essential oil contact with these pathogens results in microorganism deactivation and a formulation of carvacrol with tetracycline hydrochloride was previous successfully used for the treatment of local mouth bacterial infections and candidiasis [8]. Further horizons could be established by the combinations of carvacrol or *L. pubescens* EO with classic antibiotics for the treatment of enteric pathogens.

## 3. Materials and Methods

### 3.1. Plant Material

Flowering aerial parts of *Lavandula pubescens* and different organs (leaves, stems, ripe and unripe fruits) of *Juniperus procera* were collected at Wadi Thee Ghazal, Near Taif, Makkah Province (GPS coordinates 21°05′56.1″ N 40°20’33.1″ E), in June. Flowering aerial parts of *Pulicaria incisa* ssp. *candolleana* were collected at Jabal Al-Lawz, Tabuk province (GPS Coordinates 28°51′18.1″ N, 35°23′22.6″ E), in November. Plants were photographed (Figure 1) and voucher specimens were deposited in the herbarium of the pharmacognosy lab, Umm Al-Qura University (*L. pubescens*, LP-EOM/SA-IT; *J. procera* JP-EOM/SA-IT; *P. incisa* ssp. *candolleana* PIC-EOM/SA-IT).

### 3.2. Chemicals and Reagents

Solvents (*n*-hexane HPLC grade, dimethyl sulfoxide (DMSO) analytical grade and carvacrol were purchased from Sigma–Aldrich (St. Louis, MO, USA). Mueller Hinton Agar was purchased from HiMedia Laboratories Pvt, Ltd. (Mumbai, India). Ciprofloxacin was purchased from Acros (New Jersey, USA), vancomycin and amikacin paper discs were purchased from Bioanalyse (Ankara, Turkey), vancomycin hydrochloride was kindly gifted by Hikma (Amman, Jordan).

### 3.3. Essential Oil Extraction

The air-dried plant material was finely crushed and subjected to EO hydrodistillation in a Clevenger-type apparatus for 2 h. Aliquots of the obtained EOs were diluted to 10% in HPLC grade *n*-hexane prior to GC-MS injection, while the remaining parts were stored in freezer at −18 °C until antimicrobial testing.

### 3.4. Gas Chromatography-Mass Spectrometry Analyses and Peak Identification

Gas chromatography-electron impact mass spectrometry (GC-EIMS) analyses were performed with an Agilent 7890B gas chromatograph (Agilent Technologies Inc., Santa Clara, CA, USA) equipped with an Agilent HP-5MS (Agilent Technologies Inc., Santa Clara, CA, USA) capillary column (30 m × 0.25 mm; coating thickness 0.25 µm) and an Agilent 5977B single quadrupole mass detector (Agilent Technologies Inc., Santa Clara, CA, USA). The oven temperature program was set to rise from 60 °C to 240 °C at 3 °C/min. Temperatures were set as follows: injector temperature, 220 °C; transfer-line temperature, 240 °C. The carrier gas was He, at 1 mL/min flow. The acquisition was performed with the following parameters: full scan, with a scan range of 35–300 *m/z*; scan time: 1.0 s; threshold: 1 count. The identification of the constituents was based on the comparison of their retention times (t_R_) with those of pure reference samples and of their linear retention indices (LRIs), which were determined relatively to the t_R_ of a series of *n*-alkanes (C9–C25). The detected mass spectra were compared with those listed in the commercial libraries NIST 14 and ADAMS, as well as in a homemade mass-spectral library, built up from pure substances and components of EOs of known composition and MS literature data [44,50].

### 3.5. Diffusion Assay on Agar Plates

As recommended by the National Committee for Clinical Laboratory Standards (NCCLS manual), the antimicrobial activity of EOs of the investigated plants was assayed by the diffusion method. The tested bacterial strains were: *E. faecalis*—ATCC 51299, *E. faecalis* (Vancomycin-resistant Enterococci, VRE), *S. epidermidis*—ATCC 12228, *S. aureus*—ATCC 43300, *S. aureus* (Methicillin-resistant Staphylococcus aureus, MRSA) 43300, *S. typhimurium*—ATCC 14028, *A. baumannii*, (Carbapenem-resistant Enterobacteriaceae, CRE)—ATCC 19605, *S. sonnei*–25931, *K. pneumonia* (Extended spectrum beta-lactamases, ESBL)—ATCC 700603, *K. pneumonia* (CRE)—ATCC 1705, *P. aeruginosa*, *Proteus mirabilis*—ATCC 43071, and *E. coli*—ATCC 35218. Above acronyms are as follows: ATTC: American Type Culture Collection; *VRE*: Vancomycin-resistant Enterococci; MRSA: Methicillin-resistant Staphylococcus aureus; ESBL: Extended spectrum beta-lactamases; CRE: Carbapenem-resistant Enterobacteriaceae. Each bacterial strain was suspended in Mueller–Hinton Broth and adjusted to 0.5 McFarland scale turbidity. The surface of Muller–Hinton agar plates was swabbed in three directions with standard inoculum, using sterile cotton swabs. The plates were allowed to dry for 10 min before 3-mm wells were cut into the Muller–Hinton agar using sterile plastic pipettes. Then, the wells were filled with 20 µL and 30 µL of EO dissolved in DMSO at the rate of 1/10 *v/v*, since the EO of *Lavandula pubescens* was the only active one, the test was repeated by using the pure major constituent carvacrol at the concentration of 10 mg/mL in dimethyl sulfoxide (20 mg of carvacrol was dissolved in 2 mL of DMSO), 30 µL per well (corresponding to 300 µg per well), with vancomycin (30 µg disc) as a positive control for Gram-positive bacterial strains while amikacin (30 µg disc) and ciprofloxacin dissolved in DMSO with the rate 1000 µg/1000 µL (30 µg per well) were used as a positive control for Gram-negative bacterial strains; furthermore, dimethyl sulfoxide was used as a negative control. All plates were incubated at 37 °C for 24 h under aerobic conditions. After the incubation period, the plates were examined, and the diameter of each inhibition zone was measured.

### 3.6. Microdilution of Broth Assay

The microdilution method, using 96-well microtiter plates according to the Clinical and Laboratory Standards Institute (CLSI) guidelines [16], was conducted to evaluate the antibacterial activity. Performance standards for antimicrobial susceptibility testing were based on the 18th informational supplement of CLSI document Wayne (PA Clinical Laboratories Standards Institute, pp. 46–52) [9].

### 3.7. Determination of the Minimum Inhibitory Concentration (MIC) and Minimum Bactericidal Concentration (MBC)

MICs and MBCs of *L. pubescens* EO, carvacrol, ciprofloxacin, and vancomycin hydrochloride were determined with the broth micro-dilution method, with sterile 96-well microtiter plates for the determination of the MIC and MBCs of the tested samples. All samples were dissolved in DMSO, with exception of vancomycin hydrochloride which was dissolved in water. Basically, the first column in microtiter plates contained 200 µL of EO, carvacrol, or antibiotics and the other subsequent wells contained 100 µL of Mueller–Hinton broth. EO, carvacrol and antibiotic were serially diluted by transferring 100 µL to the next well to produce serial dilutions. Mueller–Hinton broth (100 µL containing the bacteria 0.5 McFarland) was added to each well containing 100 µL of the tested EO, carvacrol or antibiotics. Sterilized Mueller–Hinton broth alone was used as the negative control, and bacterial broth with dimethyl sulfoxide (DMSO) was only used as control. The microdilution plates were incubated at 37 °C overnight. The MIC was determined by selecting the lowest concentration of sample that completely inhibited the growth of the organism and compared with the growth control (Table 4). Wells with no visible growth in MIC were sub-cultured using 10 μL of the selected wells and placed on Muller–Hinton agar plates. The MBC was determined by taking 10 μL of the selected column and placing it on the Mueller–Hinton agar plates as well. All plates were incubated for 24 h at 37 °C and the colony forming units (CFUs) were counted. MIC was determined by selecting the lowest concentration of the tested sample that completely inhibited the visible growth of a microorganism after overnight incubation in the well. The MBC was defined as the lowest concentration of the sample that prevents any growth of an organism after being sub-cultured on the Mueller–Hinton agar plate [9].

## 4. Conclusions

This study proposed the use of aromatic wild-growing species of Saudi Arabia as potential, natural sources of bioactive antimicrobial agents. As available and exploitable biomass, local wild-growing species can represent a promising source of new bioactive natural compounds, especially in the light of alternatives to traditional medicinal compounds, towards which antibiotic-resistance is a scary but growing phenomenon. Moreover, their availability and the facility of the proposed extraction method (hydrodistillation) constitute a remarkable cost reduction compared to existing antimicrobial agents.

*L. pubescens* and *J. procera* are abundantly distributed in the high region of Asir and Hijaz mountains, so they could be a source for mass production of essential oils, especially after the successful plantation in that region, while *P. incisa* subsp. *candolleana* belongs to an important genus, rich in essential oil with potential antimicrobial properties [51,52].

Our results encourage us to continue investigation into the possible mechanism of action of carvacrol, especially against *A. baumannii*, which is causing an increasing number of deaths in vulnerable patients. In addition, the EO of *L. pubescens* can be further studied for its use as an alternative natural approach to lowering the spread of infectious diseases during large gatherings and pilgrimages, such as Hajj. Furthermore, it could be of interest to verify a possible synergistic effect between the pure constituents of EOs and a number of antibiotics.

## Figures and Tables

**Table 1:** Complete chemical composition of all the hydrodistilled essential oils.

Constituents.	l.r.i. ^a^	l.r.i. ^b^	Relative Abundance (%)					
			***L. pubescens***	***P. incisa***	***J. procera***	***J. procera***	***J. procera***	***J. procera***
			Aerial Parts	Aerial Parts	Leaves	Stems	Unripe Fruits	Ripe Fruits
santolina triene	910	908	- ^c^	-	-	-	0.1	-
tricyclene	928	926	-	-	-	0.4	0.1	-
α-thujene	933	931	-	0.1	-	0.3	-	-
α-pinene *	941	939	-	1	33.9	62.5	31.4	31.3
α-fenchene	954	951	-	-	1.6	0.5	1.2	1.1
camphene *	955	953	-	0.2	0.6	0.7	0.3	0.5
thuja-2,4 (10)-diene	959	957	-	-	-	0.3	-	-
sabinene *	977	976	-	-	-	0.1	0.2	-
β-pinene *	982	980	-	-	4.6	3.4	3.6	3.3
2,3-dehydro-1,8-cineole	992	991	-	2.3	-	-	-	-
myrcene *	993	991	3.5-	-	3.7	3.3	4.1	4.2
*cis*-dehydroxylinalool oxide	1002	999	-	0.2	-	-	-	-
α-phellandrene *	1006	1005	0.2					
δ-3-carene *	1013	1011	0.3	-	30.3	7.3	26.8	25.8
α-terpinene *	1020	1018	0.2					
*p*-cymene *	1028	1027	0.3	0.5	0.5	0.4	0.2	0.2
limonene *	1032	1031	0.2	0.2	2.8	2.3	2.1	2.4
*(Z)*-β-ocimene *	1042	1040	4.1	-	-	-	-	-
*(E)*-β-ocimene *	1052	1050	0.4	-	-	-	-	-
γ-terpinene *	1063	1062	-	0.2	-	-	0.1	0.3
*cis*-linalool oxide (furanoid) *	1076	1074	-	1.6	-	-	-	-
terpinolene *	1089	1088	6.1	-	3.8	1.6	5.8	4.9
*trans*-linalool oxide (furanoid) *	1090	1088	-	1.0	-	-	-	-
*p*-cymenene	1091	1089	-	-	-	0.7	-	-
linalool *	1101	1098	-	33.0	0.3	0.4	0.1	-
isopentyl-2-methylbutanoate *	1102	1099	-	2.6	-	-	-	-
α-cyclocitral	1117	1116	-	0.4	-	-	-	-
α-isophorone *	1120	1118	-	0.3	-	-	-	-
chrysanthenone	1126	1123	-	10.3	-	-	-	-
α-campholenal	1127	1125	-	-	-	0.6	-	-
*trans*-pinocarveol *	1141	1139	-	-	-	1.1	-	-
camphor *	1145	1144	-	-	-	-	-	0.2
*trans*-pinocamphone	1162	1160	-	-	-	0.2	-	-
*cis*-chrysanthenol	1163	1162	-	8.0	-	-	-	-
pinocarvone	1164	1162	-	-	-	0.4	-	-
borneol *	1168	1165	-	-	-	0.1	-	-
4-terpineol *	1179	1177	-	0.9	-	0.3	-	-
*p*-cymen-8-ol *	1185	1183	0.7	-	-	0.2	-	-
α-terpineol *	1191	1190	0.1	0.7	0.5	1.2	0.3	0.2
myrtenal *	1194	1193	-	-	-	0.5	-	-
myrtenol *	1195	1194	-	-	-	0.3	-	-
verbenone *	1207	1204	-	0.3	-	0.2	-	-
8,9-dehydrothymol	1221	1221	-	0.2	-	-	-	-
methylcarvacrol *	1244	1244	13.4	-	-	-	-	-
*cis*-chrysanthenyl acetate	1264	1262	-	1.3	-	-	-	-
isopiperitenone	1271	1272	-	1.8	-	-	-	-
bornyl acetate *	1287	1285	-	-	0.5	0.7	0.7	0.1
*p*-menth-1-en-9-ol	1294	1291	0.2	-	-	-	-	-
carvacrol *	1298	1298	55.7	-	-	-	-	-
eugenol *	1358	1356	-	8.9	-	-	-	-
*(E)*-β-damascenone	1382	1380	-	0.3	-	-	-	-
β-bourbonene	1385	1384	-	-	2.0	-	-	-
β-elemene	1392	1391	-	-	0.4	-	0.2	0.2
*(E)*-jasmone	1393	1390	-	0.4	-	-	-	-
*(Z)*-jasmone *	1395	1394	-	5.7	-	-	-	-
β-caryophyllene *	1419	1418	3.8	1.1	2.9	1.6	5.9	6.4
dimethoxy-*p*-cymene	1424	1423	-	0.2	-	-	-	-
α-humulene *	1455	1454	0.1	-	3.2	1.5	6.7	6.9
*(E)*-β-farnesene	1459	1458	-	-	-	0.5	-	-
γ-muurolene	1478	1477	-	-	-	0.4	-	0.1
germacrene D	1482	1480	-	-	1.6	0.2	6.3	5.8
thymyl isobutyrate	1490	1489	-	2.1	-	-	-	-
neryl isobutyrate *	1492	1491	-	0.3	-	-	-	-
valencene *	1493	1491	-	-	-	0.1	-	-
viridiflorene	1495	1493	-	-	-	-	-	0.1
α-muurolene	1499	1499	-	-	-	-	-	0.1
germacrene A	1505	1503	-	-	-	-	-	-
α-bulnesene	1507	1505	-	-	-	-	0.2	0.2
β-bisabolene	1508	1509	9.1	2	-	9.1	-	-
*trans*-γ-cadinene	1514	1513	-	0.7	-	-	-	0.2
δ-cadinene	1524	1524	-	-	0.2	0.4	0.3	0.4
elemol	1550	1549	-	-	3.0	0.1	1.2	1.3
germacrene B	1557	1556	0.1	-	-	-	-	0.3
germacrene-D-4-ol	1574	1574	-	-	-	-	0.1	0.2
caryophyllene oxide *	1582	1581	1.1	1.9	0.5	0.6	-	0.3
cedrol *	1601	1599	-	-	-	1.7	-	-
humulene epoxide II	1607	1606	-	-	0.4	0.5	-	0.3
*10-epi*-γ-eudesmol	1619	1619	-	-	-	-	-	0.2
γ-eudesmol	1632	1630	-	-	0.5	-	0.1	0.3
T-cadinol	1641	1640	-	1.4	-	-	0.1	0.3
β-eudesmol	1650	1649	-	1.1	-	-	0.1	0.3
α-cadinol	1652	1653	-	0.3	-	-	-	-
α-eudesmol	1653	1652	-	-	-	-	0.4	1.2
Monoterpene-hydrocarbons			15.3	2.2	81.8	83.8	76.0	74.0
Oxygenated-monoterpenes			70.1	64.2	1.3	6.0	1.1	0.5
Sesquiterpene-hydrocarbons			13.1	3.8	10.3	4.7	19.6	20.7
Oxygenated-sesquiterpenes			1.2	4.7	4.4	2.9	2.0	4.4
Phenylpropanoids			-	8.9	-	-	-	-
Apocarotenes			-	1.0	-	-	-	-
Non-terpene-derivatives			-	8.7	-	-	-	-
Total-identified (%)			99.6	93.5	97.8	97.4	98.7	99.6
Extraction yield (% *w/w*)			1.09	1.14	0.51	<0.1	2.7	2.39

^a^ Linear retentions index on a HP5-MS capillary column; ^b^ values from the literature [44,45]; * comparison with authentic standards; ^c^ not detected.

**Table 2:** Antimicrobial evaluation of essential oils (EOs) in agar diffusion assay (200 µg/well), R = Resistant.

Microbial Strains	*L. pubescens* Aerial Parts EO	*P. incisa* Aerial Parts EO	*J. procera* Leaves EO
*Enterococcusfaecalis* ATCC * 51299	12 mm	R	R
*Enterococcusfaecalis(VRE)* ATCC 51299	R	R	R
*Staphylococcus aureus* ATCC 25923	R	R	R
*Staphylococcus aureus* (MRSA) ATCC 43300	R	R	R
*Staphylococcus epidermidis* ATCC 12228	10 mm	R	R
*Salmonella typhimurium* ATCC 700720	13 mm	R	R
*Klebsiella pneumonia* (ESBL) ATCC 14028	R	R	R
*Klebsiella pneumonia* (CRE) ATCC 1705	R	R	R
*Acinetobacter baumannii* (CRE) ATCC 19605	15 mm	R	R
*Shigella sonnei* ATCC 25931	11 mm	R	R
*Pseudomonas aeruginosa* ATCC 15442	R	R	R
*Proteus mirabilis* ATCC 3071	R	R	R
*Escherichia coli* ATCC 35218	R	R	R

* ATCC: American Type Culture Collection; VRE: Vancomycin-resistant Enterococci; MRSA: Methicillin-resistant Staphylococcus aureus; ESBL: Extended spectrum beta-lactamases; CRE: Carbapenem-resistant Enterobacteriaceae.

**Table 3:** Antimicrobial evaluation of carvacrol in dimethyl sulfoxide (DMSO) 10% *w/v* and *L. pubescens* EO in DMSO 10% *v/v* in agar diffusion assay. R = Resistant. Data expressed as (mean ± SD) of two replicates.

Tested ATCC Strains	Diameter of Zone of Inhibition (mm)						
	Carvacrol 300 µg/well	*L. pubescens* EO 200 µg/well	*L. pubescens* EO 300 µg/well	Ciprofloxacin 30 µg/well	Amikacin 30 µg (Disc)	Vancomycin 30 µg (Disc)	DMSO
*Enterococcus faecalis* ATCC 51299	12 ± 0.00	12 ± 0.00	14 ± 0.00	-	-	18.5 ± 0.71	R
*Staphylococcus epidermidis* ATCC 12228	15 ± 0.00	10 ± 0.00	15 ± 0.00	-	-	28 ± 0.00	R
*Salmonella typhimurium* ATCC 700720	20 ± 0.00	13 ± 0.00	19 ± 0.00	29 ± 0.00	20 ± 0.00	-	R
*Acinetobacter baumannii (CRE)* ATCC 1605	15 ± 0.00	15 ± 0.00	24 ± 0.00	9 ± 0.00	15 ± 0.00	-	R
*Shigella sonnei* ATCC 25931	15 ± 0.00	11 ± 0.00	16 ± 0.00	30 ± 0.00	R	-	R

**Table 4:** Determination of the Minimum Inhibitory Concentration (MIC) and Minimum Bactericidal Concentration (MBC) expressed in µg/mL of carvacrol, *L. pubescens* EO, ciprofloxacin and vancomycin hydrochloride; data expressed as (mean ± SD) of two replicates.

Tested ATCC Strains	Carvacrol		*L. pubescens* EO		Ciprofloxacin		Vancomycin	
	MIC	MBC	MIC	MBC	MIC	MBC	MIC	MBC
*Enterococcus faecalis* ATCC 51299	500 ± 0.00	1000 ± 0.00	312 ± 0.00	625 ± 0.00	-	-	0.06 ± 0.00	0.12 ± 0.00
*Staphylococcus epidermidis* ATCC 12228	500 ± 0.00	1000 ± 0.00	312 ± 0.00	625 ± 0.00	-	-	0.06 ± 0.00	0.12 ± 0.00
*Salmonella typhimurium* ATCC 700720	250 ± 0.00	500 ± 0.00	78 ± 0.00	156 ± 0.00	0.70 ± 0.17	1.4 ± 0.65	-	-
*Acinetobacter baumannii* ATCC 1605	250 ± 0.00	500 ± 0.00	78 ± 0.00	156 ± 0.00	15 ± 0.00	30 ± 0.00	-	-
*Shigella sonnei* ATCC 25931	250 ± 0.00	500 ± 0.00	78 ± 0.00	156 ± 0.00	0.70 ± 0.17	1.4 ± 0.65	-	-

## Data Availability

Data is contained within the article.

## Sample Availability

Samples of the compounds are available from the authors.
